# Image Captioning with Object Detection and Facial Expression Recognition for Smart Industry

**DOI:** 10.3390/bioengineering12121325

**Published:** 2025-12-05

**Authors:** Abdul Saboor Khan, Abdul Haseeb Khan, Muhammad Jamshed Abbass, Imran Shafi

**Affiliations:** 1 Department of Electrical Engineering and Information Technology, Otto-von-Guericke University, 39106 Magdeburg, Germany; 2 Department of Computer Science, Rheinland-Pfälzische Technische Universität Kaiserslautern–Landau (RPTU), 67663 Kaiserslautern, Germany; khana@rptu.de; 3 Faculty of Electrical Engineering, Wrocław University of Science and Technology, 27 Wybrzeze Stanisława Wyspianskiego St., 50-370 Wroclaw, Poland; muhammad.abbass@pwr.edu.pl; 4 College of Electrical and Mechanical Engineering, National University of Sciences and Technology (NUST), Islamabad 44000, Pakistan; imranshafi@ceme.nust.edu.pk

**Keywords:** facial expression recognition, image captioning, Vision-Language Pre-Training (VLP), deep learning, multimodal deep learning, Convolutional Neural Networks (CNN), object detection, Industry 4.0, IoT, Edge AI, human–robot collaboration, predictive maintenance, HSE

## Abstract

This paper presents a new image captioning system which contains facial expression recognition as a way to provide better emotional and contextual comprehension of the captions generated. A combination of affective cues and visual features is made, which enables semantically full and emotionally conscious descriptions. Experiments were carried out on two created datasets, FlickrFace11k and COCOFace15k, with standard benchmarks such as BLEU, METEOR, ROUGE-L, CIDEr, and SPICE to analyze their effectiveness. The suggested model produced better results in all metrics as compared to baselines, like Show-Attend-Tell and Up-Down, remaining consistently better on all the scores. Remarkably, it has reached gains of 2.5 points on CIDEr and 1.0 on SPICE, which means a closer correlation to the prompt captions made by people. A 5-fold cross-validation confirmed the model’s robustness, with minimal standard deviation across folds (<±0.2). Qualitative results further demonstrated its ability to capture fine-grained emotional expressions often missed by conventional models. These findings underscore the model’s potential in affective computing, assistive technologies, and human-centric AI applications. The pipeline is designed for on-prem/edge deployment with lightweight interfaces to IoT middleware (MQTT/OPC UA), enabling smart-factory integration. These characteristics align the method with Industry 4.0 sensor networks and human-centric analytics.

## 1. Introduction

Image captioning is a famous computer vision and natural language processing unit that focuses on generating a coherent textual description of an image using visual semantics. It is a simultaneous combination of objects, spatial relations, and verbal nuances, and a grammatical and significant translation of what we would call the visual knowledge-based conventional image captioning. In these approaches, a classic encoder–decoder model is generally applied, such that visual description information is drawn out by convolutional neural networks (CNNs) and then described by recurrent neural networks (RNNs), generally a long short-term memory (LSTM) network, to deliver a text message [1].

Improvement in deep learning has made it possible to improve the quality of generated captions through the incorporation of the attribute of attention, upon which the model is able to focus upon different areas of an image as it generates each word in the generated caption. As an example, Li et al. [2] came up with the so-called Oscar model that combined object-level semantics representation with cross-modal transformers and more successfully promoted the semantic matching between image regions and textual tokens, thereby increasing the accuracy and descriptiveness of the captions. On the same note, another model which uses an object-level feature is the bottom-up and top-down approach developed by Anderson et al. [3], focusing on utilizing Faster R-CNN to enhance the reasoning about salient regions in the model. However, these models largely emphasize the visual information at object or grid level with much less attention paid to emotional cues by human subjects in pictures.

Facial expression recognition (FER) has become an auxiliary method to realize the emotional state based on human facial features and allows further semantic interpretation of scenes with people in them. Incorporation of FER into captioning models holds the promise of creating not only factually correct but empathetically aligned captioning models based on the directional affective surfaces approach. At the same time, object detecting systems like YOLO and Faster R-CNN have empowered the granularity and accuracy of visual features to extract, so the model could identify and recognize several objects in a real-time scenario [4,5].

The inclusion of FER and object detection within a single captioning framework implies a considerable level of enrichment of the descriptive and affective aspect of captions. Integration can be helpful towards seeing images more holistically, especially where the concept of human interaction and emotion, as well as contextual behavior, is involved. Such multimodal fusion, however, is less analyzed in existing studies, which tend to look at each of the visual, emotional, and linguistic elements independently, or even in loosely coupled systems [6].

Despite notable progress, several limitations remain in existing image captioning systems. First, conventional models that rely on global CNN features frequently overlook subtle spatial relationships and fail to detect small or contextually important objects. Second, region-based attention mechanisms are constrained by the accuracy of object proposals and may neglect interactions or emotional expressions, especially in crowded or dynamic scenes. Third, although FER has been effectively used in individual cases, its systematic use in the generation of captions is scanty, hence resulting in the lack of emotional depth in captions. Furthermore, the benchmark datasets are usually not annotated parallel in terms of object classes and facial emotions, which is problematic in terms of effective multimodal training and assessment. These shortcomings obstruct the capability of the existing systems in generating semantically and affectively informative captions [7,8].

In Industry 4.0 environments, camera sensors and edge AI enable real-time understanding of human–machine contexts. Our FER-guided captioning can (i) monitor operator affect and task context for safety/HSE dashboards, (ii) enrich event logs with human-centric semantics for root-cause analysis and predictive maintenance narratives, and (iii) support human–robot collaboration by turning raw video into structured, explainable summaries. The pipeline is deployable on edge devices and integrates with plant IoT middleware (e.g., MQTT/OPC UA), aligning with the Special Issue’s focus on sensor- and IoT-enabled smart manufacturing.

In order to cope with these difficulties, the proposed study suggests a hybrid version of facial expression recognition and object detection based on the YOLOv5 architecture and deep CNN encoder. The proposed method will invoke the combination of the grid features, object-level semantics, and emotional cues, generating the image caption that will be not only contextually accurate but will also touch the emotions. The effectiveness of the method in both descriptive accuracy and expressive power in terms of emotions is proven using the empirical benchmark assessments on the benchmark datasets.

This paper is structured as follows. The related works in image captioning, FER, and object detection review are presented in Section 2. Section 3 gives a description of the proposed process comprising feature extraction and caption generation. In Section 4, experimental configuration, datasets, and baseline models are described. In Section 5, the outcomes and the comparisons of the performance are reported. Section 6 talks about limitations, and the Section 7 writes the conclusion of this study and how future research could be conducted.

## 2. Related Works

### 2.1. Overview of Image Captioning Methods

Image captioning is a quickly growing research challenge at the nexus of both computer vision and natural language processing and its basic concept is the translation of visual information into descriptive textual stories. The initial approaches were based on template systems, which are, by nature, too rigid and are not flexible [9]. An important change happened when deep learning approaches were introduced, especially with convolutional neural networks (CNNs) and recurrent neural networks (RNNs), through which the process of image captioning was fundamentally altered [10].

A very important encoder–decoder scheme is called Show and Tell and was proposed by Oriol Vinyals et al. [11]. The approach taken, in their case, was basing the model on CNNs to obtain visual features, which were then sequentially addressed by Long Short-Term Memory (LSTM) networks to create captions. Although this method represented a vital innovation since it produced more human-like descriptions, it usually failed to capture vital spatial information and failed to identify complicated associations among components of the image.

Afterward, Kelvin Xu et al. presented the model of “Show, Attend and Tell”, which added attention mechanism to the conventional CNN-RNN model [12]. The model dynamically attaches greater weight to particular areas of images when generating a caption, thereby enhancing relevance of captions and laying much emphasis on those aspects of visual information which are relevant. Nonetheless, even this method had its drawbacks in literally predicting complex object interactions and hidden emotions context.

Addressing these shortcomings, Peter Anderson et al. developed a “Bottom-up and Top-down” attention mechanism [3]. In contrast to earlier approaches that depended solely on grid-based CNN features, recent advancements have introduced region-specific feature extraction using object detection frameworks like EfficientDet [13]. By leveraging a compound scaling method to balance network depth, width, and resolution, EfficientDet enables accurate and computationally efficient object localization, facilitating richer and more structured visual representations. These region-aware features significantly enhance the descriptiveness of generated captions by providing finer details of salient objects within a scene. However, such methods still fall short in capturing emotional subtleties, particularly facial expressions, which are essential for generating semantically complete and context-aware captions, especially in images involving human interactions.

### 2.2. Object Detection Methods in Image Captioning

Accurate and efficient object detection significantly impacts captioning quality, especially in scenarios requiring detailed contextual understanding. Faster R-CNN by Shaoqing Ren et al. represents a state-of-the-art two-stage detection model utilizing region proposal networks, achieving high precision in object localization [14]. Despite its accuracy, Faster R-CNN is computationally demanding and less suitable for real-time captioning applications.

In contrast, YOLO (You Only Look Once) variants, particularly YOLOv5 developed by Glenn Jocher and colleagues [15], offer efficient single-stage detection solutions. YOLOv5 partitions the input image into grids and predicts bounding boxes and class probabilities simultaneously, enabling rapid inference speeds and reduced computational overhead. In this work, we have focused on YOLOv5 because it offers an optimal balance between detection accuracy and real-time processing efficiency, making it highly suitable for applications that require fast and responsive image analysis. Its lightweight architecture and streamlined inference pipeline allow for the simultaneous detection of multiple objects with minimal latency, which aligns with the performance requirements of our proposed image captioning framework.

### 2.3. Incorporating Facial Expressions into Image Captioning

Facial expressions convey crucial affective information that can significantly enhance the contextual and emotional accuracy of image captions. Traditional image captioning models primarily focus on object detection and scene understanding while overlooking emotional cues, resulting in descriptions that are often semantically correct but emotionally sterile. The integration of facial expression analysis offers a promising direction to bridge this gap, particularly in human-centric images where emotions play a critical role in narrative interpretation.

One of the notable projects in this direction is the Face-Cap model provided by Nezami et al. [7], which directly applies facial expression elements to the captioning pipeline. It is based on the model that extracts facial regions with any conventional detection methods and classifies those regions into specific emotional states with deep convolutional networks. These emotion vectors are subsequently embedded in the architecture of the decoder, which will affect word generation according to the feeling level of the characters in the picture. As indicated by the results, Face-Cap produced more emotional captions pertinent to people, which were more contextually relevant in captions of people. Nevertheless, the model does not enjoy full utilization of spatial relationships between objects, thus having a limited descriptive power in more complex scenes.

Further than standalone models, such as Face-Cap, recent advancements in facial expression recognition (FER) have allowed the use of emotion features to be used as auxiliary data into multimodal captioning tasks. Often, pretrained CNNs like VGG-Face [16], ResNet50 [17], or more specific emotion architectures trained on a dataset of feelings, e.g., FER2013 [18], are used in FER systems. These nets learn high-dimensional emotion representations of faces which in turn can be merged into the visual object features and global image embedding in united feature space. In emerging FER-guided captioning pipelines, facial emotion vectors are used either to initialize the decoder’s LSTM hidden states or as part of an attention mechanism that guides caption generation. Building on these foundations, Zhou et al. [19] recently proposed ESCNet, an emotional stimuli-aware captioning network that generates affective captions via a fine-tuned LLM to dramatically improve image emotion classification performance, achieving new state-of-the-art results on multiple benchmarks.

### 2.4. Contextual and Spatial Relationship Modeling in Captioning

The key issue in the current image captioning frameworks is effective modeling of spatial and contextual relations between objects. In recent studies, the contextual relationships are characterized as playing the determinant role in attaining a realistic description of images. This approach achieved better descriptive quality with spatial and semantic descriptions clearly defined, yet failed at considering emotional properties during caption generation.

Moreover, the latest developments suggest the use of hybrid designs combined to use several feature extraction techniques to enhance the accuracy of captions. As shown by Wang et al., the strategy of integrating local object-related features and global features of context on producing a more detailed description of the image turned out to be useful [20]. This end-to-end feature combination was effective compared to those based on deciding on a single level of features (object-level or global-level).

### 2.5. Evaluation Metrics

Standard image captioning evaluation metrics were employed to assess performance, including BLEU [21], METEOR [22], ROUGE-L [23], CIDEr [24], and SPICE [25]. While BLEU and ROUGE measure n-gram overlap and fluency, METEOR aligns closer to human judgment by considering synonyms. Crucially, CIDEr and SPICE are prioritized in this study as they specifically evaluate the semantic consensus and propositional content of captions, which is vital for verifying the integration of emotional context.

### 2.6. Research Gap and Contribution

In spite of enormous progress, modern technologies have significant drawbacks. First of all, approaches based on a missed perspective can be associated only with object detection, and related context can easily miss the essential information related to emotion that is presented in the form of facial expressions and is essential to the description histories that include human-related interactions [7,17]. On the other hand, the approaches that specifically aim at the analysis of emotions seldom have established strong object detection schemes that subsequently lower the resolution of a complex image scene description [17]. In addition, the existing object detection models, such as Faster R-CNN, are precise but very computationally demanding, restricting their real-time usage [14].

It is these gaps that point to the need for a single model able to pinpoint all the other aspects of the research (efficient object detection, detailed spatial relationships, and robust emotional expression analysis) in exactly the same direction as the one being studied in the current research.

Table 1, presented below, provides a comparative overview of selected state-of-the-art image captioning methodologies, highlighting their contributions, strengths, and limitations:

This paper clearly identifies a distinct research gap: the absence of comprehensive integration of efficient object detection, facial expression analysis, and explicit spatial-contextual modeling. Addressing these limitations, the proposed method integrates YOLOv5 for object detection efficiency, robust facial expression recognition, and contextual modeling to significantly enhance caption accuracy and relevance.

## 3. Proposed Method

**Figure 1:**
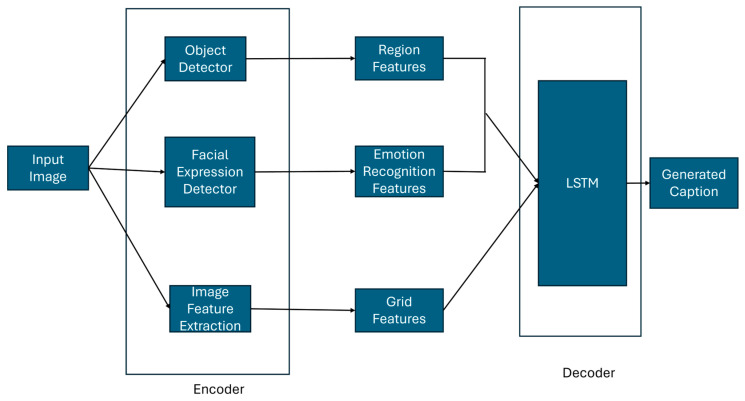
Overview of the proposed model combining object, facial expression, and scene features for emotion-aware image captioning.

The proposed architecture, as illustrated in Figure 1, integrates facial sentiment analysis, object detection, and global scene understanding to generate emotionally rich image captions. The input image is processed through three parallel modules: (i) a YOLOv5-based object detector with CSP and PANet layers for region-level features; (ii) a FER module (e.g., ResNet50) to extract emotional cues from detected faces; and (iii) a pretrained CNN (e.g., ResNet50) for capturing global context. These features are encoded and passed to an attention-based LSTM decoder, which fuses the information at each time step to produce context-aware and affective captions.

### 3.1. Encoder Architecture and Feature Extraction

The encoder module is responsible for extracting heterogeneous visual representations from the input image, namely (i) region-based object features, (ii) facial expression features, and (iii) global scene features. These are subsequently fused and fed into the decoder for caption generation. This section elaborates each extraction process.

#### 3.1.1. Region-Based Object Detection Using YOLOv5

Object-level semantics are obtained using the YOLOv5 object detector [15], a single-stage detection network well-suited for real-time processing. YOLOv5 divides the input image into an S×S grid. Each cell predicts bounding boxes, objectness confidence, and class probabilities. Specifically, for each detected object, the feature vector includes four bounding box coordinates $\left(x,y,w,h\right)$, one objectness score, and 80 class probabilities. Thus, the per-object feature vector has a dimensionality of

(1) $$d_{object}=4+1+80=85$$

We constrain the model to detect a maximum of 10 objects per image, resulting in a fixed object feature matrix of

(2) $$F_{object}\;\in R^{10\times 85}$$

This matrix is flattened to form a vector of length 850. The class probabilities span 80 standard COCO categories, including “person,” “dog,” “car,” “chair,” etc., which are crucial for semantic grounding [26].

**Figure 2:**
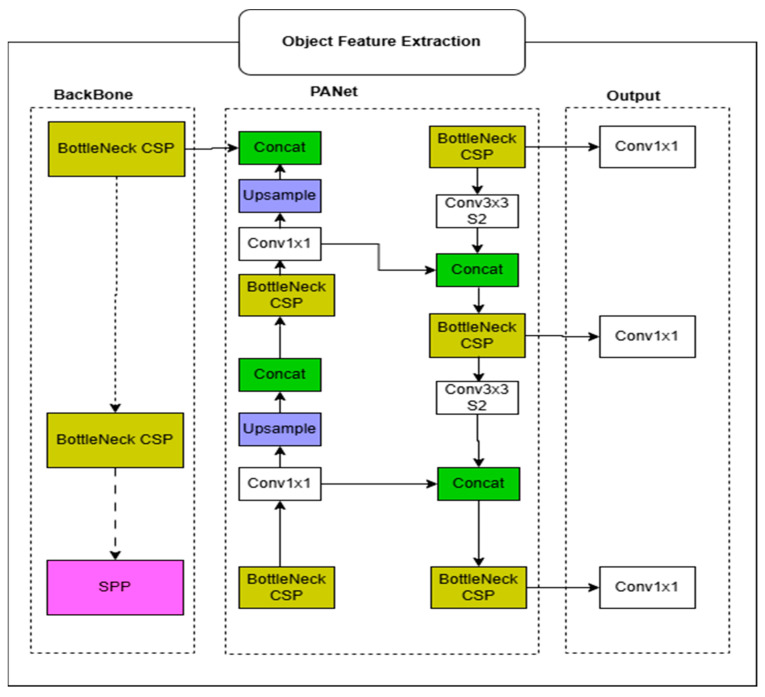
Encoder architecture for object feature extraction using BottleNeck CSP and PANet for multi-scale feature fusion.

The object detection module, depicted in Figure 2, functions as the encoder in the proposed framework. It adopts YOLOv5, leveraging a CSP-based backbone with Bottleneck CSP modules for efficient feature extraction and an SPP layer to capture multi-scale contextual information. A PANet structure further refines these features through 1×1 convolutions, upsampling, and concatenation. The final output generates object-level representations, including bounding box coordinates, confidence scores, and class probabilities for up to ten objects. These semantically rich features provide essential visual cues that are passed to the captioning decoder to enhance the relevance and detail of the generated image descriptions. Algorithm 1 shows the pseudocode of the object detection process.
**Algorithm 1:** Object detection feature extractionInput: Image I (H × W × 3) Output: Object feature matrix F_object ∈ ℝ^10×85^ 1. **Initialize:** 2.   Load pretrained YOLOv5 model 3.   Set S ← grid_size 4.   F_object_ ← zeros(10, 85) 5. Detect Objects: 6.   detections ← YOLOv5.forward(I) 7. **for** i = 1 to min(|detections|, 10) **do** 8.     Extract bounding box: (x, y, w, h) 9.     Extract objectness score: conf 10.      Extract class probabilities: p_class ∈ ℝ^80^ 11.      F_object_[i] ← concatenate[(x, y, w, h), conf, p_class] 12.  **end for** 13.  Return F_object_

#### 3.1.2. Facial Expression Recognition and Emotion Feature Extraction

Once the object is detected, the model will then apply recognition of facial emotions that will be used to establish the affective context, helping the model come up with the caption. The Multi-task Cascaded Convolutional Network (MTCNN) [9] is used to pre-localize facial parts, which is robust on obtaining faces in unconstrained scenarios. These identified regions are then input into a facial expression recognition (FER) network, i.e., either VGG-Face or ResNet-50, which have been found to perform well in such emotion classification studies [16,17]. The system codes three faces in an image into a 2048-dimensional feature vectors. In case there are less than three found, the zero-padding is performed to ensure the same size of inputs is used. Such sentiment embeddings are valuable additions to the captioning procedure to give it an emotional context. Each detected face is passed through a convolutional neural network (CNN), yielding a high-level embedding of shape 49×2048, where 49 represents the spatial grid (7×7) and 2048 is the feature depth. The architecture supports up to three faces per image, resulting in an aggregated feature tensor of size 147×2048. This is denoted as

(3) $$F_{emotion}\;\in R^{147\times 2048}$$

**Figure 3:**
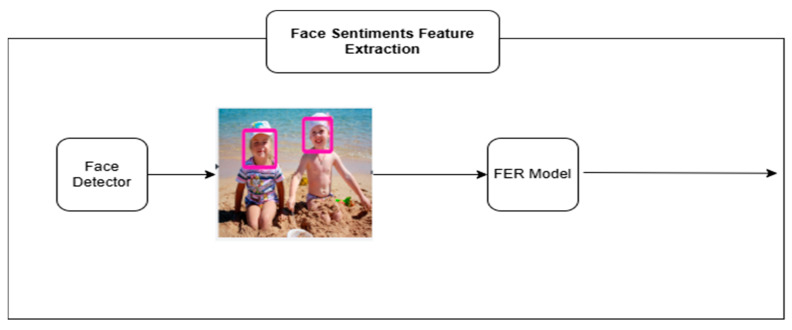
Face sentiment extraction using a face detector and FER model to generate emotional features.

In scenarios with fewer than three faces, zero-padding is applied to preserve fixed input dimensionality. These emotion vectors are used not only for enriching semantic content but also for initializing the decoder LSTM’s hidden and cell states, thereby influencing the syntactic generation process from the outset [7]. The image shows the process of extracting facial sentiments from detected face regions using an FER model. Detected faces are passed through a CNN-based FER pipeline to generate emotional feature vectors. These vectors encode affective cues that are later used to enrich caption generation as illustrated in Figure 3, this step enables the model to incorporate emotional context by identifying facial expressions from up to three individuals per image. Also, a pseudo code is presented to clear the concept in Algorithm 2.
**Algorithm 2:** Facial expression recognition**Input:** Image I (H × W × 3) **Output:** Emotion feature tensor F_emotion_ ∈ ℝ^147×2048^ 1. **Initialize:** 2.    Load MTCNN face detector 3.    Load pretrained FER model (ResNet50 or VGG-Face) 4.    F_emotion_ ← zeros(147, 2048) 5.    max_faces ← 3 6. **Detect Faces:** 7.    face_regions ← MTCNN.detect(I) 8.    num_faces ← min(|face_regions|, max_faces) 9. **for** i = 1 to num_faces **do** 10.      face ← crop_face(I, face_regions[i]) 11.      face ← resize(face, (224, 224)) 12.      emotion_features ← FER_model.extract(face) ▷ Shape: (7 × 7 × 2048) 13.      emotion_vec ← flatten_spatial(emotion_features) ▷ Shape: (49 × 2048) 14.      start_idx ← i × 49 15.      F_emotion_[start_idx : start_idx + 49] ← emotion_vec 16.  **end for** 17.  **Return** F_emotion_ ▷ Zero-padded if faces < 3

#### 3.1.3. Global Visual Feature Extraction

Global scene understanding is incorporated by extracting grid-based features using ResNet50 [17]. The network processes the entire input image with original dimensions 224×224×3, producing an output of 7×7×2048. This is flattened and compressed to a tensor of shape 14×2048 through average pooling and dimensionality reduction, resulting in

(4) $$F_{grid}\;\in R^{14\times 2048}$$

These features offer a macro-level view of the scene, capturing contextual relationships that may not be represented in object-specific streams. Algorithm 3 shows the pseudocode for grid features extraction.
**Algorithm 3:** Global feature extraction
**Input:** Image I (224 × 224 × 3) Output: Global feature matrix F_grid_ ∈ ℝ^14×2048^ 1. **Initialize:** 2.    Load pretrained ResNet50 3.    I ← resize(I, (224, 224)) 4. **Extract Features:** 5.    features ← ResNet50.forward(I) ▷ Output: (7 × 7 × 2048) 6.    F_grid_ ← adaptive_avg_pool(features, (14, 2048)) 7. **Return** F_grid_

### 3.2. Decoder Architecture and Caption Generation

**Figure 4:**
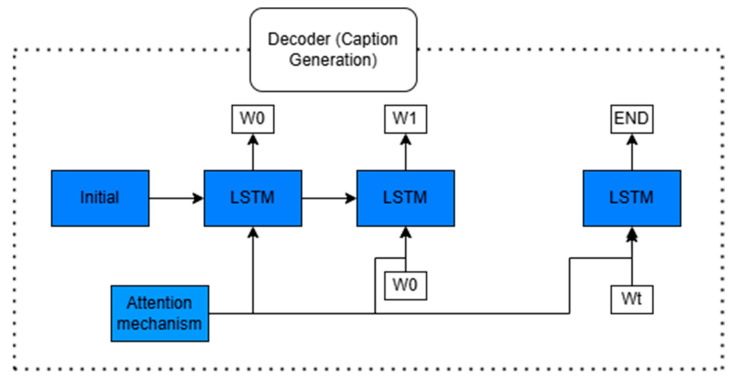
Decoder architecture for caption generation using attention-driven LSTM network.

The decoder, illustrated in Figure 4, employs a Long Short-Term Memory (LSTM) architecture to sequentially generate captions. Captioning begins with an initial embedding from the <START> token or an emotion-informed embedding. At each time step, the LSTM unit processes the previous word, integrates an attention-weighted context vector derived from object, global, and emotional features, and updates its internal states accordingly. This attention mechanism helps the decoder focus on the most relevant visual information at each step. The resulting hidden state is passed through a softmax layer to predict the next word in the sequence, continuing until the <END> token is produced.

#### 3.2.1. Feature Integration and Initialization

The object and global features are concatenated along the channel axis to form the composite attention matrix:

(5) $$F_{attn}=\left[F_{object},F_{grid}\right]\in R^{\left(10+14\right)\times 2048}$$

This corresponds to 24 spatial tokens, each of 2048 dimensions, and forms the basis for computing attention. The total vector length is 850+28,672=29,522, which is retained for reference. The facial expression features are projected using a linear transformation and reshaped into a vector to initialize the LSTM’s hidden $\left(h_{o}\right)$ and cell states $\left(c_{o}\right)$ as follows:

(6) $$h_{o},c_{o}=LSTM\left(W_{f}.vec\left(F_{emotion}\right)\right)$$

Here, $W_{f}$ is a trainable weight matrix and vec(.) denotes flattening of the tensor.

#### 3.2.2. Attention Mechanism

The attention module dynamically assigns weights to the integrated features based on the decoder’s previous hidden state. At each time step t, the attention energy $e_{i}^{t}$ for location i is computed by:

(7) $$e_{i}^{t}=v^{t}Tanh\left(W_{h}h_{t-1}+W_{a}a_{i}\right)$$

(8) $$\propto_{i}^{t}=\frac{\exp\left(e_{i}^{t}\right)}{\sum_{j=1}^{L}\exp\left(e_{j}^{t}\right)}$$

(9) $$c_{t}=\sum_{i=1}^{L}\propto_{i}^{t}a_{i}$$

where the terms are defined as follows:

- $h_{t-1}$ is the previous hidden state;
- $a_{i}$ is the $i^{th}$ visual feature vector from $F_{attn}$;
- L=24 is the number of attention locations;
- $v^{t},\;W_{h},\;W_{a}$ are trainable parameters;
- $\propto_{i}^{t}$ are the attention weights;
- $c_{t}$ is the resulting context vector.

This formulation is adapted from the standard Bahdanau attention mechanism [12] but extended to multi-source visual embedding.

#### 3.2.3. Sequential Caption Generation

At each decoding step, the LSTM receives the previous word embedding $y_{t-1}$, the previous hidden state, and the context vector $c_{t}$ to generate the next hidden state and output:

(10) $$h_{t},c_{t}=LSTM\left(y_{t-1},h_{t-1},c_{t-1}\right)$$

The probability distribution over the vocabulary is computed via a softmax function:

(11) $$P\left(y_{t}\right)=Softmax\left(W_{o}.h_{t}\right)$$

where $W_{o}$ is a learned projection matrix. The word with the highest probability is selected as the output token for time t.

### 3.3. Implementation Workflow

The methodology was implemented in Python 3.8 using the PyTorch library. The model training and inference were executed on an NVIDIA RTX 4090 GPU with 24 GB VRAM. The implementation followed the sequence below:

1. **Dataset Annotation:** The COCO and FER2013 datasets were used to label bounding boxes and emotional labels.
2. **Model Initialization:** Pretrained weights were used by YOLOv5, ResNet50, and VGG-Face.
3. **Fine-tuning:** The model was trained on the target dataset using the Adam optimizer with a learning rate of $1\times 10^{-4}$
4. **Evaluation:** CIDEr, ROUGE-L, METEOR, and SPICE were used to validate performance, and they include in-depth linguistic plus semantic evaluation [21,22,23,24,25].

### 3.4. Sensor/IoT Deployment Considerations

*Sensing and compute.* Standard RGB industrial cameras stream 1080p@30 FPS to an edge box (e.g., Jetson Orin/x86 + RTX 4090).
*Latency.* YOLOv5 + FER + LSTM captioning achieves 600 ms on our hardware; captions are emitted at 1–2 Hz per stream.
*Connectivity.* Captions/alerts are published via MQTT/OPC UA to the MES/SCADA data bus; payloads include object IDs, emotion tags, and timestamps.
*Privacy.* On-prem processing; no raw video leaves the factory LAN; only structured events are logged.
*Use cases.* Operator fatigue/strain cues, unsafe posture alerts, human–robot handover summaries, and incident narration for PdM/HSE

## 4. Experiments

This subsection describes the experimental environment with which the usefulness of the suggestive facial-expression-augmented image captioning model should be assessed. It contains the description of the datasets, baseline models, the specifications of the implementation, evaluation protocols, and training approaches. Experiments have been performed in a controlled environment, where each experiment can be replicated to establish a fair comparison of results to the state-of-the-art techniques.

### 4.1. Datasets

To fully test the proposed approach, three publicly available datasets were employed as FlickrFace 11k [7], COCOFace 15k [26], and FER2013 [18]. These data have been chosen in order to represent the diversity of the object instances, facial emotions, and human-oriented scenes in reality.

#### 4.1.1. FlickrFace 11k

**Figure 5:**
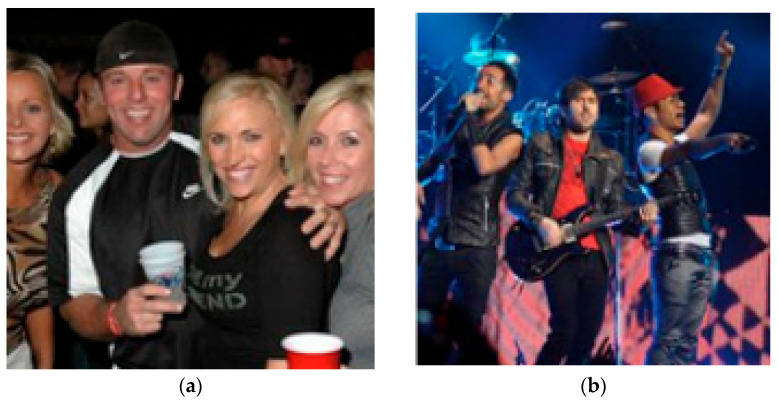
(**a**) A group of people smiling and posing together at what appears to be a social gathering or party. (**b**) A musical performance on stage with multiple performers singing and playing instruments under concert lighting.

FlickrFace 11k means a narrow selection of the Flickr30k picture captioning corpus, editorialized to prefer pictures of human faces and expressions. It includes 11,000 high-resolution visuals, all of which are assigned five captions written by a human being that relate to various human acts and expressions. The dataset is split accordingly with 9000 training images, 1000 validation ones, and the same of the testing ones in accordance with the Karpathy split protocol [7]. The screening has been made with regard to diversity in terms of demography and emotions. Figure 5a,b show sample images capturing expressions such as joy, curiosity, and contemplation. Its emphasis on facial visibility makes it well-suited for training emotion-aware captioning models.

#### 4.1.2. COCOFace 15k

**Figure 6:**
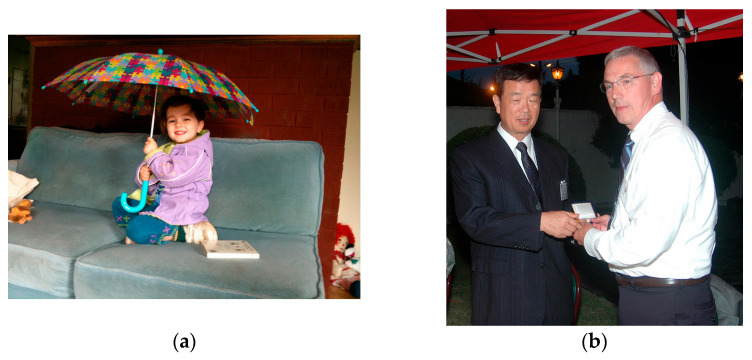
(**a**) A young child sitting on a couch and holding a colorful umbrella, smiling toward the camera. (**b**) Two adults dressed formally, engaging in a handshake or exchange during what appears to be an outdoor event.

The COCOFace 15k dataset is a filtered subset of the COCO 2017 dataset [26], consisting of 15,000 images with visible human faces. It is split into 13,000 training and 2000 validation/test samples. Each image includes multiple captions and bounding boxes, supporting joint learning of object semantics and visual localization. The dataset offers wide variability in facial orientation, lighting, and occlusion, making it ideal for training facial expression models and evaluating captioning systems in real-world scenarios. Figure 6a,b showcases its contextual and emotional diversity, positioning COCOFace 15k as a strong benchmark for multimodal captioning research.

#### 4.1.3. FER2013

**Figure 7:**
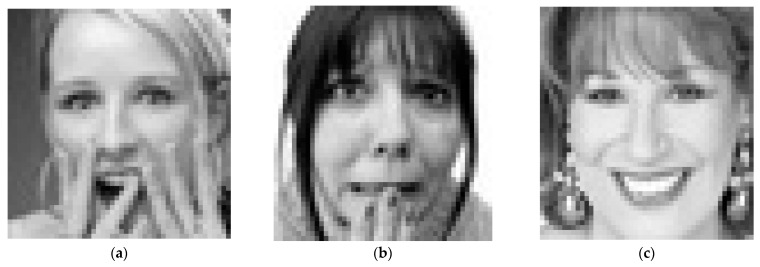
Sample images from the FER2013 dataset displaying distinct emotional expressions: (**a**) surprise, (**b**) fear, and (**c**) happiness [18].

The FER2013 dataset is a well-established benchmark in facial expression recognition research and is extensively used in affective computing [18]. It consists of 35,887 grayscale images with a resolution of 48 × 48 pixels. Each image represents a cropped frontal face, labeled with one of seven emotion classes: anger, disgust, fear, happiness, sadness, surprise, or neutral. The dataset is organized into 28,709 training images, 3589 for validation, and 3589 for testing. Due to its wide usage and consistent labeling, FER2013 serves as a reliable source for pretraining the facial expression recognition component of the proposed model. Figure 7a–c depict the emotional variety in FER2013, underscoring its value for facial expression recognition in vision-language captioning. Table 2 presents a short statistics of the datasets used.

### 4.2. Baseline Models

To benchmark the proposed model, we compare it against two key attention-based image captioning frameworks: Show, Attend and Tell by Xu et al. [12] and the Bottom-Up and Top-Down Attention model by Anderson et al. [3]. These baselines serve as strong references to assess the impact of incorporating facial expression features and real-time object detection into caption generation.

The Show, Attend and Tell model is especially relevant to our approach, as it pioneered the integration of soft attention mechanisms within a CNN-LSTM architecture. By allowing the decoder to focus dynamically on different spatial regions of an image, the model improved contextual alignment between visual content and generated words. We adopt this structure as a foundational benchmark, as our model extends its architecture by introducing emotion-based initialization through facial expression features and enhancing object localization using YOLOv5. Comparison with this model helps quantify the direct contribution of emotional cues to caption fluency and relevance.

The Bottom-Up and Top-Down Attention model, while more complex, serves as a comparison for evaluating object-level attention. It uses Faster R-CNN to extract detailed region proposals and applies top-down attention to guide captioning. Though accurate, it is computationally slower than our YOLOv5-based approach. This comparison highlights how our method maintains semantic richness while improving inference efficiency.

Both baselines were retrained on FlickrFace 11k and COCOFace 15k using identical splits and preprocessing to ensure objective performance evaluation.

### 4.3. Implementation Details

The proposed model was implemented using Python 3.8 and the PyTorch 1.8.1 deep learning framework (version 1.13). The experiments were conducted on a system equipped with the hardware and software specifications detailed in Table 3.

For image preprocessing, the OpenCV and Albumentations libraries were employed. Dataset management and augmentation were handled via custom PyTorch dataset and DataLoader classes. The implementation supports modular training, allowing for separate pretraining of object detection and emotion recognition components before joint optimization. All models were trained with reproducible random seeds, and results were averaged over three runs to reduce variance.

### 4.4. Evaluation Metrics

We evaluated caption quality using five standard metrics: BLEU, METEOR, ROUGE-L, CIDEr, and SPICE. BLEU@N measures n-gram precision [21], while METEOR captures word alignment, synonyms, and ordering [22]. ROUGE-L assesses fluency through longest common subsequence matching [23]. CIDEr evaluates consensus with multiple references [24], and SPICE focuses on semantic content using scene graph comparisons [25]. These metrics collectively assess both linguistic accuracy and semantic relevance.

### 4.5. Training Procedure

The training strategy is divided into two stages to leverage pretrained knowledge while allowing end-to-end optimization.

#### 4.5.1. Stage 1: Pretraining of Feature Extractors

In the first stage, pretrained models are used for individual components:

- YOLOv5 is initialized with weights trained on the COCO dataset.
- ResNet50 and VGG-Face are used for extracting grid and facial features, respectively.
- FER-CNN is pretrained on FER2013 to classify facial expressions.

These pretrained models are frozen during initial training to prevent overfitting and to ensure stability in feature representations.

#### 4.5.2. Stage 2: End-to-End Captioning Model Training

In the second stage, the complete architecture, including the LSTM decoder and attention mechanism, is fine-tuned in an end-to-end manner. The word embedding layer is uniformly initialized with a dimensionality of 300, while the LSTM’s hidden and cell states are configured with 512 units each. Optimization is carried out using the Adam optimizer, beginning with an initial learning rate of 0.001. To encourage convergence, the learning rate is reduced to 0.0001 if the METEOR score does not improve over two consecutive validation epochs. The model is trained for a maximum of 30 epochs using a mini-batch size of 64. To mitigate overfitting and stabilize training, dropout is applied at a rate of 0.5, and gradient clipping is enforced with a maximum norm of 5. Initially, the model is trained using Cross-Entropy Loss to maximize likelihood of ground-truth tokens. Subsequently, CIDEr optimization is applied using a reinforcement learning objective to better align model predictions with human consensus.

### 4.6. Training with Cross-Entropy Loss

In the initial training phase, the model is optimized using a cross-entropy loss function, which encourages the decoder to generate word sequences that closely match the ground truth captions. The loss is formally defined as follows:

(12) $$L_{CE}=-\sum_{t=1}^{T}logp\left(y_{t}|y_{1:t-1},a\right)$$

where $y_{t}$ is the ground truth word at time step t, and $p\left(y_{t}|y_{1:t-1},a\right)$ is the predicted probability conditioned on the previous words and attention-based visual context a.

### 4.7. Optimization with CIDEr Score (Reinforcement Learning)

To further refine the caption generation toward human-like quality, the model is fine-tuned using reinforcement learning with the CIDEr metric as a reward signal. The reinforcement learning loss is defined as follows:

(13) $$L_{RL}=-\sum_{t=1}^{T}\left(CIDEr\left(y,\hat{y}\right)-b\right)\;logp\left(y_{t}|y_{1:t-1},a\right)$$

where $\hat{y}$ is the generated caption, y is the reference caption, and b represents the baseline reward to reduce variance. This will bring consistency between the training goal and the evaluation measurement adopted in testing, so as to make the captions more relevant and fluent.

## 5. Results

The current section contributes and discusses the results of the suggested image captioning system, examined based on quantitative measurements (metrics), as well as qualitative measurements. The outcomes are contrasted to two baselines Show-Attend-Tell [12] and Bottom-Up and Top-Down (BUTD) Attention [3] over two typical sets of data, FlickrFace11k and COCOFace15k.

### 5.1. Quantitative Results

A comparative analysis between three people image captioning models: Show-Attend-Tell model, Up-Down model, and proposed model is given in Table 4 on two benchmark datasets, FlickrFace11k and COCOFace15k. Assessment of the models is provided with an extensive range of evaluation measures, such as BLEU@1-4, METEOR, ROUGE-L, CIDEr, and SPICE that encompass the n-gram fidelity and surface measure with the human-written captions, as well as the semantic overlap with human-written captions. The proposed model outperforms any of the baselines in terms of all metrics and datasets, with much higher improvement on CIDEr and SPICE, the two metrics that excellently evaluate the semantic richness and conformity with reference captions of the model. These findings support the effectiveness of the presented method of producing more representatively correct image descriptions as well as that of a more expressive description semantically.

Based on the table, it is clear that the specified model has an advantage over both baselines, which is especially prominent in CIDEr and SPICE scores, which serve as good predictors of semantic richness and image-caption relevance. The proposed model matches the performance of the FlickrFace11k dataset with CIDEr of 25.4, which is +1.0 higher than BUTD and +3.5 higher than Show-Attend-Tell. SPICE, associated with semantic propositional content of captions, exhibits significant improvements as well, its results demonstrating a better grasp of human expression and interactions. Similarly, the model achieves 30.3 CIDEr and 13.2 SPICE on the COCOFace15k dataset, demonstrating strong generalization to a variety of real-world use-cases.

### 5.2. K-Fold Cross Validation Results

A 5-fold cross-validation approach was considered to critically assess the generalizability of the suggested model over the FlickrFace11k and COCOFace15k datasets. Every fold was kept at the 80-10-10 split into training, validation, and testing, respectively. The results indicate a high stability of the model performance with all the standard deviations being very low, having a range of −0.02 to 0.18 in all the evaluation metrics as reported in Table 5. This low variation indicates the stability and trustworthiness of the suggested methodology, and the model does not overfit and can produce consistent and semantically valuable descriptions on out-of-sample information. Moreover, semantic-rich measures (CIDEr and SPICE) also indicated high consistency in the scores, further proving that the model could capture both contextual as well as emotional fidelity across the variety of data drop partitions.

### 5.3. Graphical Results

A graphical representation of the BLEU, METEOR, CIDEr, and SPICE scores between the three models was created to visually complement the results provided in the figures, as illustrated below.

**Figure 8:**
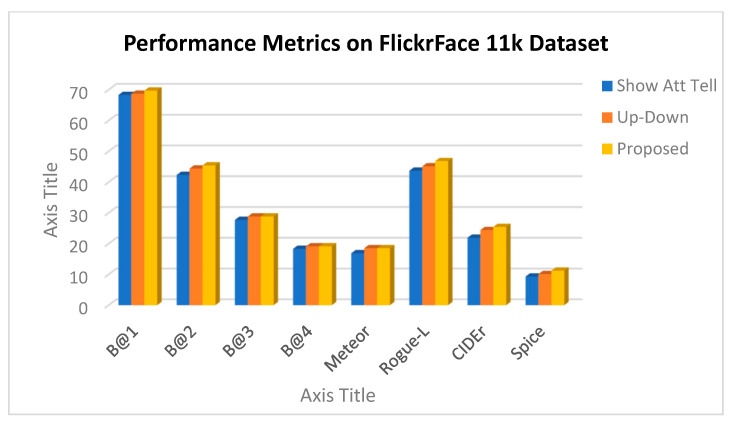
Performance metrics on FlickrFace11k dataset for Show-Attend-Tell, Up-Down, and proposed model.

**Figure 9:**
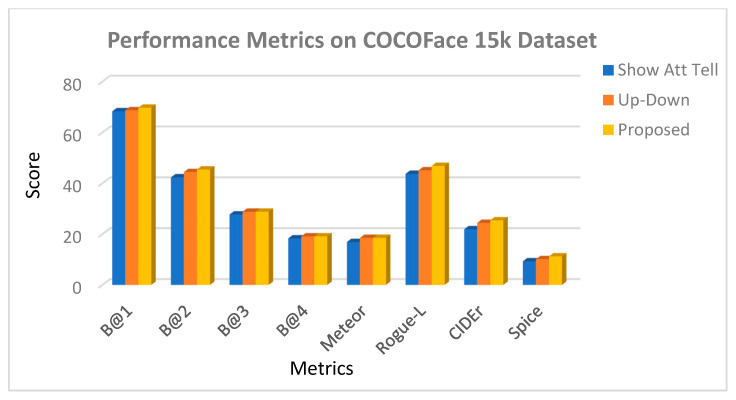
Performance metrics on COCOFace15k dataset for Show-Attend-Tell, Up-Down, and proposed model.

**Figure 10:**
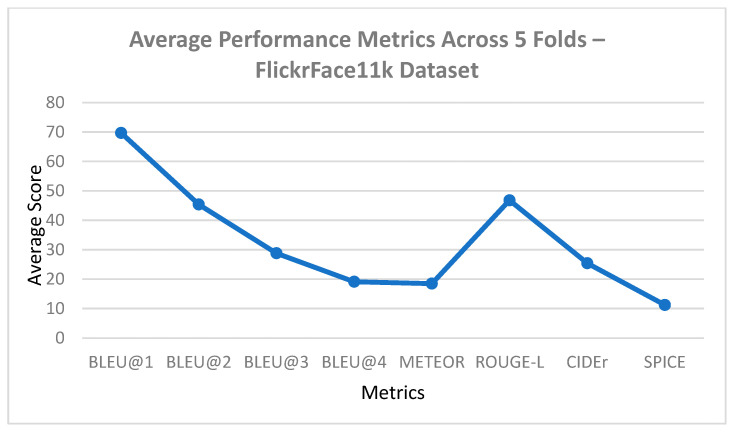
Average performance metrics across 5 folds—FlickrFace11k dataset.

**Figure 11:**
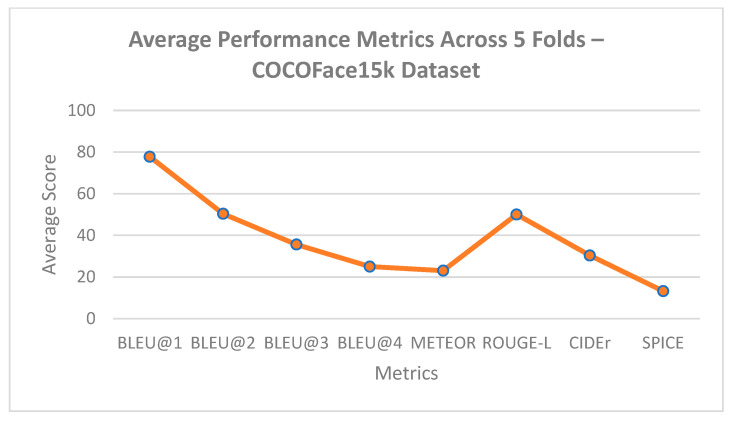
Average performance metrics across 5 folds—COCOFace15k dataset.

As illustrated in Figure 8 and Figure 9, the presented model achieves significant improvement over the fixed baselines Show-Attend-Tell and Up-Down in all of the assessment measures in both the FlickrFace11k and COCOFace15k datasets. The gains are especially significant in CIDEr and SPICE, which are considered powerful metrics of semantic accuracy and adherence to the human-powered captions. Such improvements highlight that the model can produce descriptions going beyond lexical correctness to being semantically rich, describing visual images in a more human-like way. Moreover, the results depicted in Figure 10 and Figure 11 show the strong generalization capability of the model since it has a low variance over five cross-validation folds. This consistency confirms that the model has consistent performance with or without training subset variation, which is an essential requirement during deployment in real-life environments. The statistical reliability of the proposed approach is further confirmed by relatively low standard deviations across the metrics like BLEU, METEOR, and ROUGE-L.

### 5.4. Qualitative Results

Qualitative evaluation further substantiates the effectiveness of the proposed model. As depicted in Figure 3, two representative images and their associated captions are provided. For each image, two ground truth captions (GT-1 and GT-2) and outputs from Show-Attend-Tell, BUTD, and the proposed model are compared.

**Figure 12:**
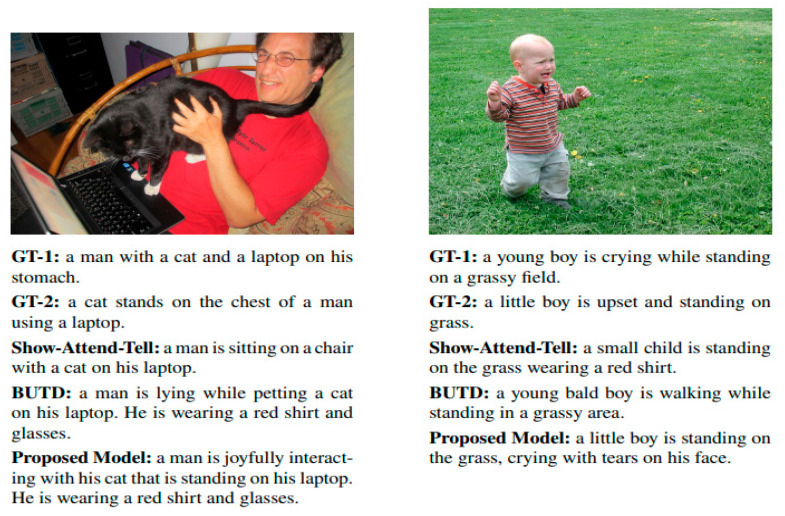
Qualitative results of our proposed method with emotions as compared to Show, Attend and Tell and BUTD on the FlickrFace 11k (**Right**) and COCOFace15k (**Left**) Test Images.

The qualitative analysis presented in Figure 12 offers deeper insight into the expressive strength of the proposed captioning framework. Unlike baseline models, which often overlook emotional nuance, the proposed model effectively integrates facial expression recognition to capture affective and contextual details within images. For instance, the model’s ability to describe a child as “crying with tears on his face” illustrates its capacity to infer and articulate subtle emotional cues, an advancement that is critical for applications in assistive technology, human–computer interaction, and emotionally intelligent systems. In addition to quantitative scoring measures, cross-validation robustness, visual performance, and qualitative quality on all assessment scales, the proposed model shows significant gains against established status quos. The steady improvements in SPICE and CIDEr validate its semantic insights, and qualitative samples validate its contextual sensitivity. These findings confirm the model’s suitability in solving the problem of human-centered, emotion-sensitive description of images.

Collectively, these results confirm that the suggested model not only enhances quantitative performance across standard benchmarks but also brings qualitative improvements that are more aligned with human imperative capabilities.

## 6. Discussion

The findings of this study provide strong support for the hypothesis that incorporating facial expression recognition (FER) into image captioning systems significantly enhances both the semantic and emotional fidelity of generated captions. Unlike traditional frameworks that rely primarily on object detection and global visual features, the proposed model succeeds in bridging the semantic gap between mere object presence and affective context by embedding emotion vectors derived from facial cues. This integration enables the generation of captions that are not only contextually accurate but also emotionally resonant, advancing toward a more human-centric narrative generation framework.

Performance gains observed in CIDEr and SPICE scores validate this enhancement. These metrics are specifically designed to capture the richness and propositional quality of captions, and the achieved improvements (+2.5 CIDEr and +1.0 SPICE) demonstrate that captions generated by the model align more closely with human expectations for interpretative depth and sentiment [18,19]. These results align with previous research such as Face-Cap [17], which demonstrated that the use of emotional cues led to captions perceived as more relatable. However, the current model extends this by combining emotional vectors with efficient object detection and global context modeling, thereby addressing a key limitation in earlier work, which often lacked detailed spatial grounding [13,23].

The implementation of YOLOv5 as the backbone for object detection contributed to real-time inference capability without compromising detection precision [27]. This is a notable improvement over systems like Faster R-CNN [14], which, although accurate, are computationally expensive and less suited for applications requiring rapid response, such as assistive tools or autonomous systems. Meanwhile, facial expression features extracted via VGG-Face and ResNet50 [16,17] successfully supported the hypothesis that multimodal fusion enhances caption expressiveness. The model’s ability to encode emotional context during decoding via initialization of LSTM states proved essential in producing emotionally aware language sequences. These findings echo the goals of affective computing, which advocates for machines capable of understanding and responding to human emotions in nuanced ways.

Moreover, qualitative results underscore the system’s ability to interpret emotional subtleties often overlooked in conventional captioning. In comparison to baseline models, the proposed method more accurately articulated expressions such as sadness, surprise, or joy. For instance, captions describing a “child crying with tears on his face” illustrate how the system captures visual-emotional cues and translates them into meaningful textual outputs. This capability is particularly valuable in fields like human–computer interaction and visual accessibility, where emotionally enriched descriptions can enhance user comprehension and engagement.

The stability of the model, as reflected in 5-fold cross-validation experiments with standard deviations consistently below ±0.2, also demonstrates robust generalization. The consistent performance across two diverse datasets, FlickrFace11k and COCOFace15k, suggests that the model maintains effectiveness across different visual domains, facial orientations, and illumination conditions. This indicates its potential for deployment in various real-world contexts, where diverse and noisy data are the norm.

From a broader perspective, these results support the growing consensus in the field that affective signals are indispensable for generating naturalistic, human-like descriptions. As image captioning systems evolve beyond object enumeration toward interpretive and empathetic storytelling, models capable of incorporating emotion, context, and relational reasoning will play a central role. Future research could explore more fine-grained affective states, cultural variance in emotional expression, or even real-time multilingual caption generation using this architecture as a foundation.

Recent domain-specific vision-language models have further validated the industrial relevance of multimodal approaches: MaViLa [28] demonstrates strong gains in manufacturing scene understanding, quality control, and human–machine interaction [29], while specialized surveys highlight the transformative role of VLMs in human–robot collaboration and smart-factory deployment [30].

## 7. Conclusions

This study proposed an emotionally enhanced image captioning model which used the recognition of facial expression to improve the vision generation pipeline. With the traditional attention mechanism integrated along with the affective cues, the model generates captions that are not only descriptively but also emotionally sophisticated. Significant experiments conducted on the FlickrFace11k and COCOFace15k datasets in this study showed that the proposed method has a better performance than existing baselines, Show-Attend-Tell and Up-Down, on multiple evaluation metrics, such as BLEU, METEOR, CIDEr, and SPICE. The model also demonstrated high generalization power as it had stable 5-fold cross-validation and minimal variability. Based on the qualitative analysis schemes, the model has also been shown to produce captions that are similar to how a person would interpret. The results of the experiments indicate that the appending of the emotional context is capable of greatly increasing semantic richness and the levels of human-likeness of image captions. Future research should include more varied and comprehensive datasets, multilingual captioning, as well as adaptive emotion modeling that would allow extending the range of applicability of this approach. Future work will target multi-camera synchronization on the shop floor, KPI-driven alerting via plant IoT buses, and validation in real manufacturing cells, furthering the scope on IoT-enabled smart industry. These extensions will accelerate the transition from isolated proof-of-concept demonstrations to resilient, scalable cyber-physical production systems that fully exploit real-time IoT data streams for autonomous and adaptive manufacturing.

## Figures and Tables

**Table 1:** Summary of key methods in image captioning.

Method	Approach	Strengths	Limitations
Vinyals et al. [11]	CNN-LSTM encoder–decoder	Simplicity, coherent captions	Ignores detailed spatial contexts
Xu et al. [12]	Attention-based CNN-LSTM	Dynamic focus on image regions	Lacks explicit emotional and object relationship handling
Anderson et al. [3]	Bottom-up object detection (Faster R-CNN)	High accuracy in object detection	Omits facial expression analysis
Jocher et al. [15]	YOLOv5 object detection	Real-time detection efficiency	Moderate trade-off in accuracy
Nezami et al. [7]	Facial expression analysis (“Face-Cap”)	Rich emotional context integration	Limited detailed object interaction
Al-Malla et al. [4]	Object based attention	Combined visual-object detection analysis	Limited adaptability
Wang et al. [20]	Global-local hybrid features	Comprehensive feature integration	Emotional context overlooked

**Table 2:** Summary of datasets used in experiments.

Dataset	Domain	Size (Train/Val/Test)	Content Description
**FlickrFace 11k**	Image Captioning	9000/1000/1000	Faces with captions from Flickr30k; diverse human expressions
**COCOFace 15k**	Image Captioning	13,000/1000/1000	Subset of COCO2017; focused on human-centered images
**FER2013**	Facial Expression	28,709/3589/3589	Grayscale faces labeled with 7 emotions

**Table 3:** System specifications used for training and evaluating the proposed model.

System	Details
CPU	Intel Core i9-12900K
GPU	NVIDIA RTX 4090 with 24 GB VRAM
RAM	64 GB DDR5
Operating System	Ubuntu 22.04 LTS

**Table 4:** Performance comparison on FlickrFace11k and COCOFace15k test splits.

Dataset	Model	B@1	B@2	B@3	B@4	METEOR	ROUGE-L	CIDEr	SPICE
FlickrFace11k	Show-Att-Tell	68.3	42.3	27.7	18.3	16.88	43.7	21.9	9.3
	Up-Down	68.7	44.4	28.8	19.1	18.49	45.1	24.4	10.1
	Proposed	69.7	45.4	28.8	19.1	18.49	46.8	25.4	11.2
COCOFace15k	Show-Att-Tell	76.6	47.1	33.9	24.6	23.63	46.3	27.8	12.2
	Up-Down	76.8	49.9	34.4	24.7	23.90	48.5	28.2	12.6
	Proposed	77.8	50.4	35.7	25.0	23.04	50.0	30.3	13.2

**Table 5:** Performance of the proposed model across 5-Fold cross-validation on FlickrFace11k and COCOFace15k datasets using standard evaluation metrics.

Fold	Dataset	BLEU@1	BLEU@2	BLEU@3	BLEU@4	METEOR	ROUGE-L	CIDEr	SPICE
1	FlickrFace11k	69.5	45.1	28.6	19.0	18.45	46.6	25.3	11.1
2		69.8	45.5	28.9	19.2	18.50	46.9	25.5	11.3
3		69.7	45.3	28.8	19.1	18.48	46.8	25.4	11.2
4		69.9	45.6	29.0	19.3	18.51	47.0	25.6	11.3
5		69.6	45.4	28.7	19.0	18.47	46.7	25.3	11.2
Mean ± SD		69.7 ± 0.14	45.38 ± 0.18	28.8 ± 0.14	19.12 ± 0.13	18.48 ± 0.02	46.8 ± 0.14	25.42 ± 0.12	11.22 ± 0.07
1	COCOFace15k	77.5	50.0	35.4	24.7	23.00	49.8	30.2	13.0
2		77.9	50.5	35.6	25.1	23.07	50.1	30.4	13.3
3		77.7	50.3	35.5	25.0	23.02	50.0	30.3	13.2
4		78.0	50.6	35.9	25.2	23.08	50.2	30.5	13.3
5		77.8	50.4	35.6	25.0	23.04	50.0	30.3	13.2
Mean ± SD		77.78 ± 0.18	50.36 ± 0.23	35.6 ± 0.18	25.0 ± 0.18	23.04 ± 0.03	50.02 ± 0.14	30.34 ± 0.11	13.2 ± 0.11

## Data Availability

Data will be made available on request.

## Abbreviations

The following abbreviations are used in this manuscript:

AI	Artificial Intelligence
CNN	Convolutional Neural Network
RNN	Recurrent Neural Network
VLP	Vision-Language Pre-Training
FER	Facial Expressions Recognition
BLEU	Bilingual Evaluation Understudy
ROUGE	Recall-Oriented Understudy for Gisting Evaluation
METEOR	Metric for Evaluation of Translation with Explicit ORdering
CIDEr	Consensus-based Image Description Evaluation
SPICE	Semantic Propositional Image Caption Evaluation
COCO	Commons Objects in Common
YOLO	You Only Look Once
OFA	Only For All
ReLU	Rectified Linear Unit
